# Factors affecting the nesting success of Swainson's Thrush (*Catharus ustulatus*) along an elevational gradient

**DOI:** 10.1002/ece3.10738

**Published:** 2024-01-17

**Authors:** Sarah C. Deckel, William V. DeLuca, Alexander R. Gerson, David I. King

**Affiliations:** ^1^ Department of Environmental Conservation University of Massachusetts Amherst Massachusetts USA; ^2^ Science Division National Audubon Society New York New York USA; ^3^ Department of Biology University of Massachusetts Amherst Massachusetts USA; ^4^ Northern Research Station USDA Forest Service Amherst Massachusetts USA

**Keywords:** abiotic factors, montane, nest survival, White Mountains

## Abstract

Montane birds experience a range of challenges that may limit their breeding success, including nest predation and severe climactic conditions. The continuing effects of climate change are causing shifts in biotic and abiotic factors that may compound these threats to montane bird species. In northeastern montane forests, many bird species are shifting downslope, potentially as the result of increased precipitation and temperature at higher elevations. Although lower elevations might be more favorable in terms of climactic conditions, nest predation is higher at lower elevations. Thus, montane birds might be faced with the opposing pressures of adverse climactic conditions at higher elevations and increased predation at lower elevations. We monitored nests of Swainson's Thrush (*Catharus ustulatus*) along an elevation gradient in the White Mountain National Forest in New Hampshire in 2016, 2018, 2019, and 2021 to examine the effect of biotic and abiotic factors on daily nest survival rate (DSR). Linear time explained the most variation of DSR in AICc model comparison, indicating that DSR decreases across the breeding season. Rain intensity (mm/h) had a weak negative effect on DSR, indicating that heavier rain per hour decreases Swainson's Thrush DSR. Moreover, we found some support for a negative interaction effect of elevation in conjunction with minimum daily temperature: DSR of Swainson's Thrush nests at low elevations (281 m) increased with increasing minimum daily temperatures and decreased at high elevations with increasing minimum daily temperatures. Our results suggest nesting survival of montane breeding birds may be at risk as heavier precipitation events become more frequent and intense due to the changing climate and raises the possibility that other passerine species could be at risk in this system.

## Graphical abstract

Montane birds experience a range of challenges that may limit their breeding success, including nest predation and severe climactic conditions. The continuing effects of climate change are causing shifts in biotic and abiotic factors that may compound these threats to montane bird species. Our results provide evidence for a potential mechanism of how climate change will affect nesting survival of montane breeding birds as heavier precipitation events become more frequent and intense, a likely outcome due to the changing climate within the White Mountains and other montane ecosystems, putting other passerine species at risk in this system.

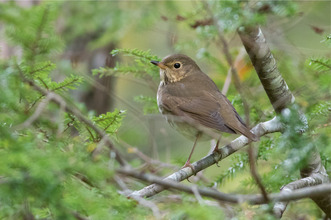

## 1 INTRODUCTION

Montane bird species that breed within high‐elevation forests are particularly vulnerable to the effects of climate change due to the narrow elevational ranges they inhabit (Scridel et al., 2018; Şekercioğlu et al., 2012). This makes them susceptible to extirpation if their elevational ranges are compressed due to climate change and anthropogenic land uses (Rodenhouse et al., 2008; Şekercioğlu et al., 2008). Research within northeastern forests of the United States confirms that the average temperature within this system has increased 0.5°C over a decade (2005–2015). Montane species within tropical systems are shifting their upper limits upslope, apparently in response to increased temperatures (Freeman et al., 2018; Neate‐Clegg, Jones, et al., 2021; Neate‐Clegg, Stuart, et al., 2021). Low‐elevation birds in the northeastern U.S. are following a similar pattern, although paradoxically montane birds are exhibiting downward elevational shifts in this system (DeLuca & King, 2017).

Regardless of their directionality, the causes of these elevational shifts, and their consequences for montane breeding birds, remains unclear. Globally, populations of montane birds are declining, potentially because elevational range shifts are exposing birds to unfamiliar resources and conditions along the elevation gradient that could affect their breeding success (Freeman et al., 2018; Lehikoinen et al., 2014; Ralston et al., 2015; Şekercioğlu et al., 2008). For example, breeding success of blackpoll warblers (*Setophaga striata*) within northeastern montane forests is lower at low elevations, likely due to increased abundance of red squirrels, a major nest predator in this system (DeLuca, 2013). Similarly, dark‐eyed juncos (*Junco hyemalis*) at high elevations in the Rocky Mountains of Canada produced nestlings in better condition and experienced higher survival rates than birds at lower elevations (Bears et al., 2009). These examples raise the possibility that lower elevations are less suitable for montane birds and that the documented downward shifts in their elevational ranges may expose these species to elevated nest predation (Boyle, 2008; Camfield et al., 2010; DeLuca, 2013).

Alternatively, abiotic factors, such as cold temperatures and increased precipitation, may affect reproductive success of birds nesting at high elevations (Pierce et al., 2019). Warmer temperatures overall contribute positively to nest success and promote larger egg size, increased clutch size, and improved hatching and fledgling success (Hargrove & Rotenberry, 2011; Johnson, 2000; Lessells et al., 2002; Martin, 1987; Reid et al., 2000; Rotenberry & Wiens, 1991). Invertebrate availability is positively related to warmer temperatures and is fundamental for a successful nest (Bears et al., 2009; Svensson & Nilsson, 1995). However, warmer temperatures may also cause predators to be more active, potentially resulting in unusually higher predation events in warmer years (Cox et al., 2013). Increased precipitation may also negatively influence nesting survival of breeding birds, particularly during intense rain events (Bordjan & Tome, 2014; Dinsmore et al., 2002; Dreitz et al., 2012; Fisher et al., 2015; McCain & Colwell, 2011; Öberg et al., 2015; Schöll & Hille, 2020; Sexson & Farley, 2012). High‐elevation sites in the northeastern U.S. are experiencing fluctuating abiotic factors, including more frequent rain events and changing temperatures, which may contribute to future population declines of breeding montane species (Karmalkar & Bradley, 2017; Wright, 2009).

Given the threats that a changing climate may impose on high‐elevation species, as well as the uncertainty as to the nature and directionality of these effects, we examined the nesting ecology of a representative montane bird species, the Swainson's Thrush (*Catharus ustulatus*) within the White Mountains of New Hampshire, USA in order to quantify the effects of weather on nesting success as well as potential interactions of these influences with elevation (Ralston & DeLuca, 2020; Ralston & Kirchman, 2013). We considered two predictions: (1) nest survival is positively related to elevation due to lower predation pressure (Boyle, 2008; Camfield et al., 2010), and (2) heavy rainfall, cold temperatures, and temperature variation (which may be more pronounced at higher elevations) negatively affects daily survival rate (Dinsmore et al., 2002; Dreitz et al., 2012; Pierce et al., 2019). Both indirect and direct effects of climate have been documented on these species' abundances (Duclos et al., 2019). Thus, investigating the abiotic effects on reproductive success of birds along elevation gradients will contribute to a better understanding of future climatic responses by species, something that is largely understudied within northeastern montane forests (Martin, 2001; Tingley et al., 2012).

## 2 MATERIALS AND METHODS

### 2.1 Study area and study species

**FIGURE 1:**
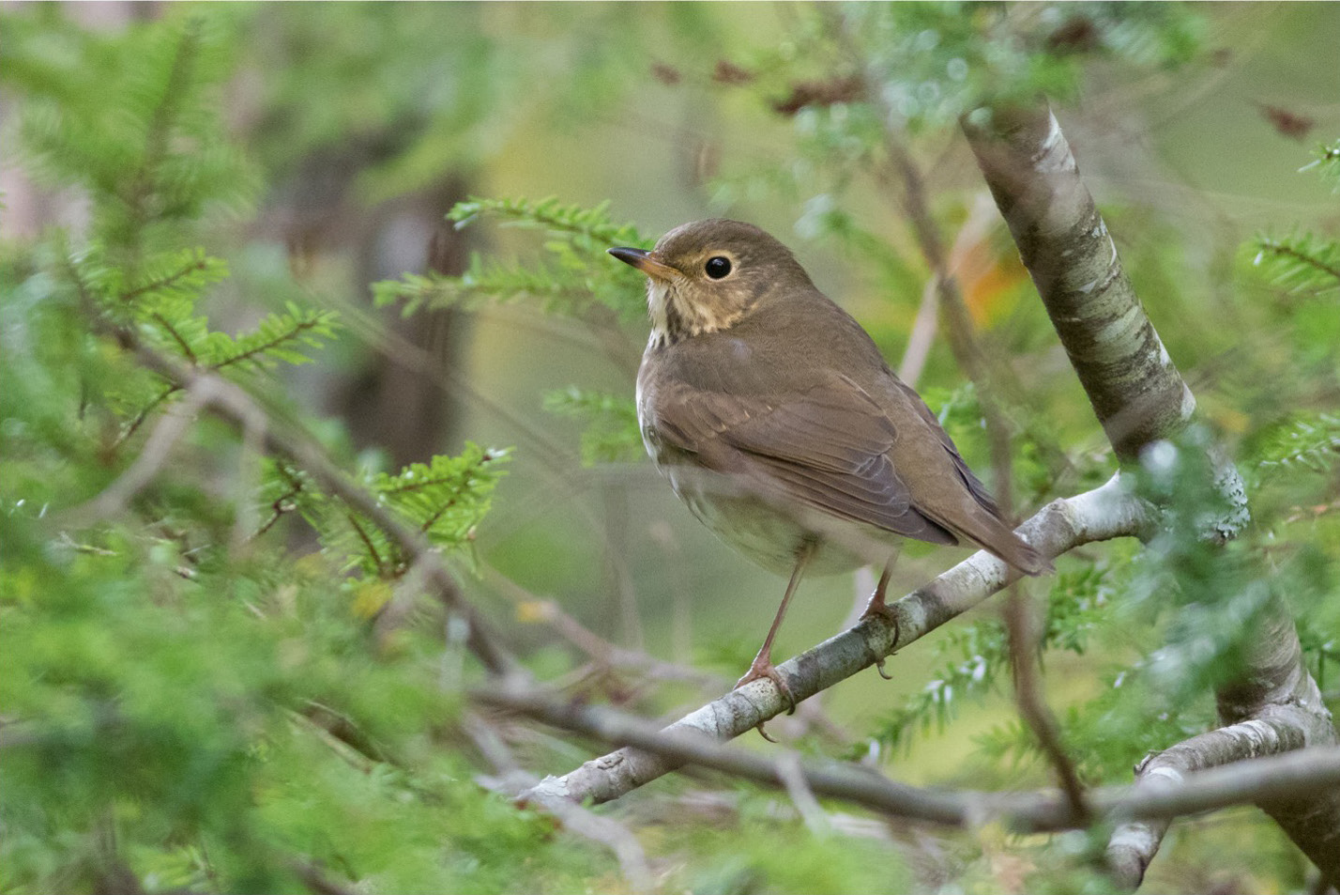
An adult Swainson's thrush (*Catharus ustulatus*) perched on a branch in the Bartlett Experimental Forest, White Mountains NH. *Photo credit: Lucas Bobay*.

We researched nest survival of Swainson's Thrush along an elevational gradient in the White Mountains of New Hampshire, USA at Mt. Jefferson (approximately 500–1250 m; 44.3045°N, 71.3176°W) and Bartlett Experimental Forest (hereafter referred to as BEF; approximately 200–300 m; 44.0556°N, 71.2973°W). The Swainson's Thrush is a Nearctic Neotropical migratory songbird which is moderately common and breeds along a wide elevation gradient within this system (Figure 1; 1200–1250 m; Mack & Yong, 2020). Paradoxically, this species has exhibited downward shifts in their elevational range over time, which may be responsible for population increases in the eastern portion of their range (DeLuca & King, 2017; Ralston & DeLuca, 2020; Ralston & Kirchman, 2013). Since this species appears to be sensitive to climate change, they are a suitable subject for this study.

Nest survival data were collected from May through August 2016, 2018, 2019, and 2021 in the White Mountains at Mt. Jefferson (all years) and BEF (2018, 2019, and 2021; field season canceled in 2020 due to COVID‐19). Forest within the study area consisted of northern hardwood forest dominated by American beech (*Fagus grandifolia*), paper birch (*Betula papyrifera*), yellow birch (*Betula alleghaniensis*), and sugar maple (*Acer saccharum*) below 700 m in elevation, a mix of deciduous and coniferous of birch trees, red spruce (*Picea rubens*), and balsam fir (*Abies balsamea*) between 700 m and 1250 m, sub‐alpine boreal tree species above 1250 m, with treeline at ~1350 m. Elevation within the BEF ranges from 200 to 400 m, though to capture low‐elevation breeding populations of Swainson's Thrush, nests were monitored only between 200 and 300 m. Tree species within BEF are primarily American beech, paper birch, red spruce, and eastern hemlock (*Tsuga canadensis*), though Swainson's Thrush typically build their nests in dense patches of small (<8 cm DBH) red spruce and eastern hemlock trees.

Swainson's Thrush typically arrive in New Hampshire between late May to early June and their nesting period is 28 days (±2) with four stages: nest building (4 days), laying and incubation of the eggs (12 days), and provisioning and caring for nestlings before fledgling (12 days). Female Swainson's Thrush typically have one brood, though they can renest, and they build their nest in red spruce, balsam fir, and paper birch trees, ranging anywhere from 3 to 10 m in height (Mack & Yong, 2020).

### 2.2 Nest monitoring

Fieldwork to locate breeding pairs and nests began approximately 25 May (±2) each year. Parental behavioral cues and rigorous nest searching are both important techniques to find nest locations (Martin & Geupel, 1993). Searching effort was relatively evenly distributed among sites. Once found, nests were marked with a GPS and flagged no <8 m away from the active nest so as not to disturb the nesting pair. We monitored each nest every 2–3 days until they were close to fledging (~11 days old), and then every day after until fledged. If presumed fledged, we searched the area meticulously to take notes such as provisioning behavior from parents (i.e., food in their bill, alarm calling, etc.) or visual sightings. A successful nest yielded at least one fledged bird, and a failed nest was depredated or abandoned. Nests were considered depredated if an active nest was observed with cracked or missing eggs, dead young nestlings (<11 days old) and/or adults, or a nest that was pulled apart. On occasion, nests were abandoned and confirmed as such if adults were absent for ≥3 visits. We collected nesting data including initiation date (i.e., date the first egg was laid), clutch size, hatch date (i.e., date the first egg hatched), and nestling condition (i.e., how old the nestlings were at the date checked and what their appearance was). When hatch date was unknown, we back‐calculated nest age based on the nestlings' condition. Finally, if nest initiation date was unknown, we backdated from known or estimated hatch date, assuming one egg was laid per day, incubation occurred for 12 days, and nestlings were at least 11 days old before fledgling (Hałupka et al., 2018). Nests were binned in four groups (BEF = 200–300 m; Low Jeff = 500–700 m; Mid Jeff = 701–900 m; High Jeff = 901–1250 m) to correspond with the matching spatial precipitation and temperature data that was collected along the elevation gradients.

### 2.3 Environmental variables

Hourly ambient temperature (°C) was collected using Thermocron® iButton loggers (Maxim Integrated) from 25 May to 31 July 2016, 2018, and 18 May to 3 August 2019 and 2021. One logger was placed at a long‐term GPS coordinate that represented the elevation gradient we were interested in and corresponded with bins used for nests (Duclos et al., 2019) (BEF = 250 m; Low Jeff = 550 m; Mid Jeff = 800 m; and High Jeff = 1200 m). Loggers were held in a Holden shield to protect the iButton from direct solar exposure (Holden et al., 2013). Daily average temperature, minimum daily temperature, and temperature range was calculated and used to analyze the effect on daily nest survival. Precipitation data were collected in 2019 and 2021 using Onset® HOBO® RG3‐M rain gauges. To capture the daily cumulative rainfall across the elevation gradient, we placed three rain gauges at Mt. Jefferson at locations similar to the iButton loggers (550 m, 800 m, and 1200 m) in open areas with no tree canopy. We used precipitation data collected in BEF from the NSF National Ecological Observatory Network (NEON), an open‐sourced data platform. Within each rain gauge is a tipping bucket that measured how much cumulative precipitation fell each day throughout the season. Precipitation data were not collected in 2016 and 2018 and, therefore, will not be analyzed with nest survival data from those years. Rain intensity was quantified by cumulative rain (mm) recorded per hour per day. We considered a 24‐h window from 12:00 am to 11:59 pm for all environmental variables. Relative proximity of temperature loggers and rain gauges to monitored nests ranged between 300 m to the closest nest, and 3 kilometers to the furthest nest, respectively. Total relative area searched at Mt. Jefferson was approximately 19 square kilometers, and 2.3 square kilometers at Bartlett Experimental Forest.

Due to equipment malfunctions from the HOBO Rain Gauge placed at 550 m (i.e., Low Jeff), precipitation data from 1 July–3 August 2021 was lost. We used Random Forest (RF) machine‐learning algorithm to estimate the missing daily precipitation at this location. When tested against other algorithms to estimate large amounts of missing precipitation data, the RF was the most preferred method in the context of our data (Aguilera et al., 2020). The RF algorithm is a nonparametric imputation method that takes observed values of each variable, and predicts the missing values, without having any assumptions about the distribution of data or imputation models. We included dummy variables to account for no rain days, a Hanssen‐Kuipers (HK) score to distinguish between occurrences and nonoccurrences of a rain event, and an NRMSE (normalized root mean squared error) to allow for the comparison of the average relative differences between the observed and imputed missing values (Aguilera et al., 2020; Hanssen, 1965). The lower the error metric NRMSE produces, the better the model performed (Moriasi et al., 2007).

Dummy variables were produced by randomly assigning the missing data matrix with presence (1) or absence (0) of rain days. For each day we considered the dates where the dummy variable was 0 and input 0 for that day, and only the days the dummy variable predicted a value of 1 did we input the numeric value estimated from the RF algorithm. The HK score ranges between −1 to +1, where 0 represents no skill or a random estimate, and 1 indicates a perfect estimate, and has been widely used to evaluate yes/no meteorological forecasts (Teegavarapu, 2013; Woodcock, 1976). We used the package *missForest* in R Studio for the RF algorithm to input missing precipitation values.

### 2.4 Statistical analysis

#### 2.4.1 Daily survival rate

We measured the daily survival rate (DSR), that is, the probability that a nest survives 1 day, to better understand how abiotic factors, nest initiation date, elevation, and other temporal variables influenced nesting survival of Swainson's Thrush (Dinsmore et al., 2002; Mayfield, 1975). We analyzed DSR using the RMark package in R Studio (Laake et al., 2013). This package calculates estimates of DSR using a maximum likelihood estimator with the logit‐link function and includes variables of interest. Assumptions for this model are that DSR was the same for all nests on all days and for all nest ages, and nest fates are independent and identically distributed (Rotella, 2006). There is currently no goodness‐of‐fit test for nest survival models in RMark, and RMark cannot run mixed models (Dinsmore et al., 2002; Laake et al., 2013; Rotella et al., 2004). Daily survival probabilities represent a constant survival rate and were calculated by obtaining the probability of success from the log‐odds estimate and raising it to the power of the number of nesting days (i.e., 28 for Swainson's Thrush). See Rotella et al. (2004) for a more in‐depth description of how MARK interprets the temporal variables as an encounter history. We included nests in our analysis when they met the following criteria: (1) known day the nest was found, (2) the last day the nest was active, (3) the last day the nest was checked, and (4) known nest fate (i.e., fledged or failed). We standardized the ordinal dates to be the duration that nests were first active (i.e., initiation date) which translated to 0 (1 June) until the last known active nest date, 63 (2 August) over the 4 years (number of occupancy days, NOCC = 63).

#### 2.4.2 Environmental variables (2019 and 2021 only)

We used cumulative daily precipitation (mm) and maximum hourly rain intensity (mm per hour) as variables in our daily survival models. Average daily temperature, minimum daily temperature, and daily temperature range (°C) were separately modeled against daily survival to better understand how temperature in the White Mountains affected daily survival probability. Nests from 2016 and 2018 were excluded from precipitation and temperature analysis because we did not collect climate data from those years. Nests were binned in the same four groups to correspond with the matching spatial precipitation and temperature data that was collected along the elevation gradient.

#### 2.4.3 Model selection

We first compared univariate models with temporal variables against DSR from all years (2016, 2018, 2019, 2021; NOCC = 63). Temporal variables included year, initiation date, seasonal time, and elevation (Table 1; models 2–7). We also included an intercept model (model 1) in which only DSR was estimated, and a quadratic time trend (model 4), which allows for nest survival to fit a curvilinear pattern, since survival over time is not always linear.

**TABLE 1:** A list of variables included in analyses of Swainson's Thrush nest survival in the White Mountains, NH.

Model name (DSR~)	Model notation
1. Constant DER	Intercept only^a^
2. Year	Year^a^
3. Linear time trend	TimeTrend^a^
4. Quadratic time trend	TT^a^
5. Nest Age	NestAge^a^
6. Elevation	Elev^a^
7. Initiation date	Init^b^
8. Daily average temperature	MeanTemp^b^
9. Daily minimum temperature	MinTemp^b^
10. Daily temperature range	TempRange^b^
11. Daily cumulative precipitation	Precip^b^
12. Rain Intensity	Intensity^b^
13. Linear time trend*Year	TimeTrendYear^b^
14. Linear time trend*Elevation	TimeTrendElev^a^
15. Elevation*Initiation Date	ElevInit^b^
16. Elevation*Rain Intensity	ElevInten^b^
17. Elevation*Daily Minimum Temp	ElevMin^b^
18. Elevation*Daily Average Temp	ElevMeanT^b^
19. Nest Age*Rain Intensity	NestAgeInten^b^
20. Nest Age*Elevation	NestAgeElev^b^
21. Elevation + TimeTrend	ElevTime^b^

*Note*: Annotations denote the year it was used: ^a^all years, ^b^2019 and 2021 only. “DSR” is the response variable, daily survival rate.

We then tested pairwise correlation comparisons within our variables (elevation, nest age, initiation date) and between the environmental variables (elevation, daily average temperature, minimum daily temperature, temperature range, daily cumulative precipitation, and rain intensity). This comparison revealed a negative correlation coefficient with elevation and daily temperature range (*p* = −.46) and, therefore, these variables were not included in the same model. We continued by building models that included temporal and abiotic variables in 2019 and 2021 (models 3–12). We also added two additional models with interaction effects (year and elevation) with seasonal time to evaluate if nest survival was affected by elevation at certain times of the breeding season (models 13 & 14). We included four more models that included biological interaction and additive effects (models 15–21) to explore all potentially important relationships between our variables and DSR.

Finally, we used AICc (Akaike's Information Criterion) to evaluate which models best described variation in DSR based on the principle of parsimony (Anderson & Burnham, 2002). The top model had an AICc value of zero, and to ensure a more rigorous selection process, we considered variables in models that were within 4 ΔAICc of the top model to be relatively similar in model fit. We considered relationships with coefficients with 90% confidence intervals not including zero to be supported (Arnold, 2010; Hein et al., 2008; Long et al., 2008). We included beta estimates, standard error (SE), confidence intervals and an odds ratio (OR) in a summarized table. To interpret effect size, we calculated the percentage of change in odds ratio by transforming the beta estimate and subtracting 1 from this value (*e* ^beta^−1). Then, we multiplied the odds ratio by 100 to determine the odds of surviving. For example, an odds ratio of 0.95 over time indicated that the odds of a nest surviving were 5% lower for every day that progressed (Allison, 2001).

## 3 RESULTS

### 3.1 Data summary

**FIGURE 2:**
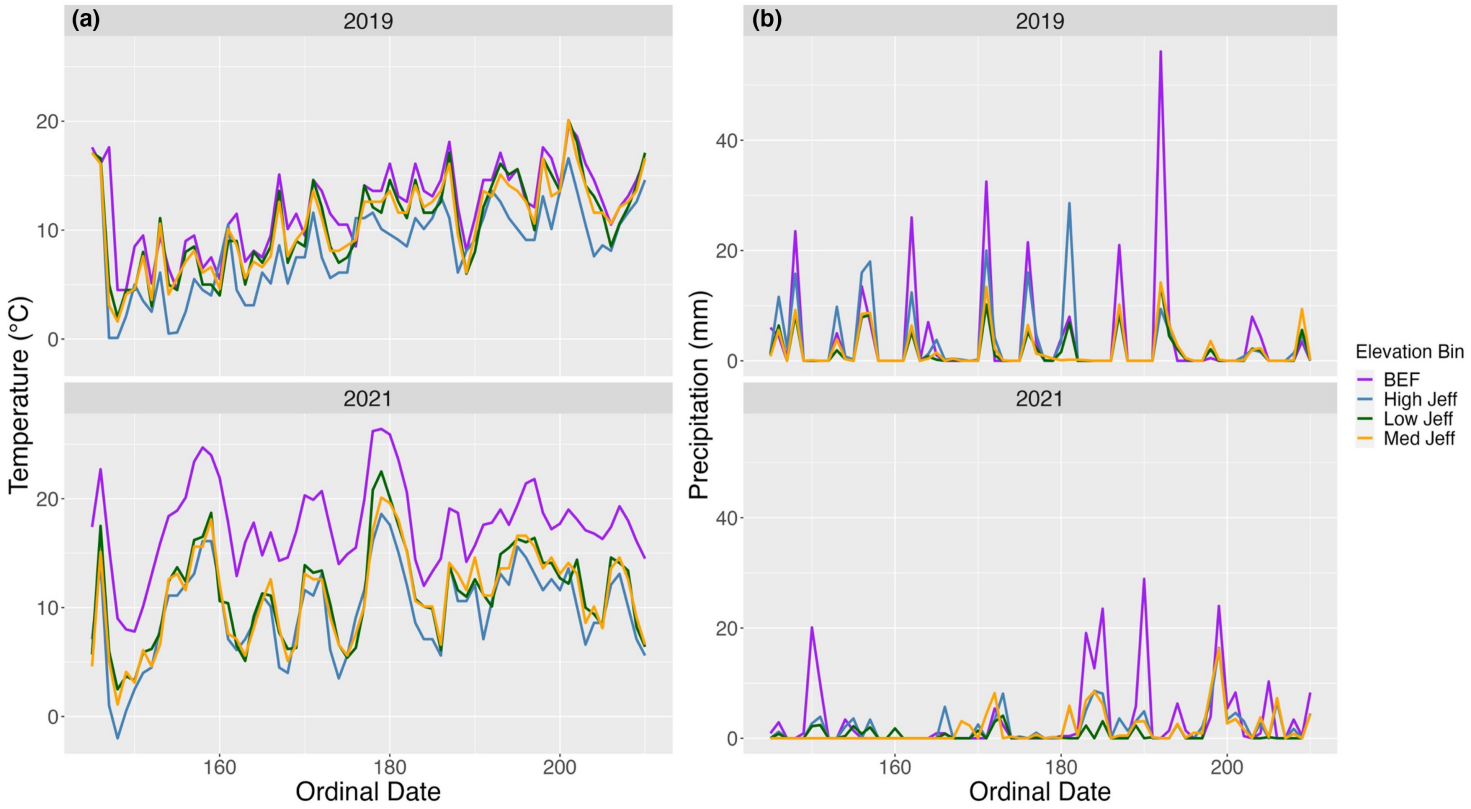
Daily minimum temperature (a) and daily cumulative precipitation (b) from 25 May–3 August 2019 and 2021 from the White Mountains, NH. Ordinal date corresponds with the day of the season (i.e., 160 = 9 June). Data is split into four elevation bins (BEF = ~250 m, Low Jeff = ~500 m, Med Jeff = ~800 m, High Jeff = ~1200 m).

A total of 58 nests were found and included in the initial stage of analyses between 2016 (*n* = 5), 2018 (*n* = 6), 2019 (*n* = 21), and 2021 (*n* = 26) from 31 May to 2 August. Out of these 58 nests, 24 succeeded (42%) and 34 nests failed (58%), either due to nest predation (71%) or abandonment (29%). We found 10 nests at BEF, 14 nests at Low Jeff, 19 nests at Mid Jeff, and 15 nests at High Jeff. Minimum daily temperature (Figure 2a) and precipitation (Figure 2b) varied among years and with elevation. Across both study sites throughout the season, average minimum daily temperature was 11.4°C (SE = 0.21), and average temperature range was 11.5°C (SE = 0.25). Forty‐four nests were included in the analysis of initiation date, precipitation, and temperature from 2019 and 2021. After backdating for initiation and hatch date, we removed 3 nests from the analysis because nests were found in the incubation stage but failed before hatch date, and thus, we could not identify initiation date. Average initiation date was June 17th (±2 days), and average clutch size was 3.1 eggs in 2019 and 3.2 in 2021. The RF algorithm calculated 34 missing days of precipitation data (NRMSE = 0.078, HK = 0.39) for the Low Jeff Rain Gauge (1 July–3 August 2021). This was 5% of our overall precipitation data collected between both sampling years. Mean, standard error and range for missing data was 1.7 (SE = 0.65) (0.0–16.3) and 2.4 (SE = 0.23) (0.0–56.1) for collected precipitation data.

### 3.2 Daily survival rate

#### 3.2.1 All years

The unadjusted daily survival probability mean for Swainson's Thrush across the four‐year sampling period was 0.95 (95% CI [0.93, 0.97]). Total probability of surviving the nesting period of 28 days based on constant DSR was 0.25 (95% CI [0.14, 0.39]). We did not find support that elevation affected daily survival probability (OR = 1.00, 95% CI [−0.001, 0.002]). There was no interaction effect between linear time and elevation (OR = 0.99, 95% CI [−0.0001, 0.00006]). AICc model comparison revealed the top model to account for the most variation in daily survival rate to be linear time (OR = 0.97, 90% CI [−0.01, 0.04]), suggesting for every day, odds of survival decrease by 3% (Table 3).

#### 3.2.2 2019 and 2021

There was no evidence that nest survival was affected by either initiation date (OR = 0.99, 95% CI [−0.02, −0.02]) or elevation (OR = 1.00, 95% CI [<−0.01, <−0.01]). Nest survival was negatively related to linear time (OR = 0.97, 90% CI [−0.01, 0.04]), indicating that nest survival declined as the season progressed. There were no interactions between elevation and initiation date (OR = 0.99, 95% CI [<−0.01, <0.01]), linear time (OR = 0.99, 95% CI [<−0.01, <0.01]) or nest age (OR = 0.99, 95% CI [<−0.01, <0.01]). Finally, we included an interaction between linear time and year because precipitation varied markedly among years (Figure 2b), but this relationship did not demonstrate an effect (Table 2; OR = 0.99, 95% CI [<−0.01, 0.01]).

**TABLE 2:** Parameter estimates, standard error (SE), odds ratio, and confidence intervals for daily nest survival for Swainson's Thrush in the White Mountains, NH for two separate models, the most supported with all years (*n =* 58; 2016, 2018, 2019, and 2021) and the most supported model with just 2019 and 2021 (*n =* 44).

Model	Beta (intercept)	Beta (*b*)	SE	Odds ratio	Lower CI	Upper CI
All years (2016, 2018, 2019, and 2021)						
*TimeTrend*	3.80	−0.03	0.01	0.97	−0.06	0.00
*TT*	3.35	<−0.01	<0.01	1.00	−0.00	0.00
**DSR**	–	2.59	0.01	–	2.54	2.62
Elev	2.59	<0.01	<0.01	1.00	−0.00	0.00
NestAge	3.04	<0.01	0.02	1.00	−0.03	0.02
2019 and 2021 only						
**ElevMin**	−2.46	−0.00	<0.01	0.99	<−0.01	<−0.01
*TimeTrend*	3.89	−0.03	0.02	0.97	−0.06	<−0.01
**Intensity**	3.12	−0.10	0.04	0.91	−0.19	−0.01
TT	3.38	<−0.01	<0.01	0.99	<−0.01	<0.01
DSR	–	2.99	0.01	–	2.54	2.62
TempRange	2.33	0.06	0.05	1.06	−0.04	0.17
**ElevMeanT**	−3.83	<−0.01	<0.01	0.99	<−0.01	<−0.01
Elev	2.74	<0.01	<0.01	1.00	<−0.01	<0.01
MinTemp	3.25	−0.02	0.06	0.97	−0.13	0.09
Init	4.29	−0.01	0.02	0.99	−0.05	0.03
MeanTemp	3.32	−0.02	0.07	0.97	−0.15	0.11
Precip	2.97	0.01	0.05	1.01	−0.09	0.12
NestAge	3.03	<0.01	0.02	1.00	−0.04	0.04

*Note*: The intercept‐only model is denoted as DSR. Supported models that have 95% CI significance and are denoted when the model's name is in bold, and models that have some support have 90% significance and are denoted in italics. Beta (*β*) estimates are reported on the logit‐scale. Values were rounded to the nearest 0.01. Refer to Table 1 for model names.

There was no relationship between DSR and average daily temperature (OR = 0.97, 95% CI [−0.07, 0.20]), minimum daily temperature (OR = 0.97, 95% CI [−0.06, 0.16]) or daily temperature range (OR = 1.06, 95% CI [−0.05, 0.16]; Table 2). There was an interaction between the effects of elevation and minimum daily temperature on DSR (OR = 0.99, 95% CI [<−0.01, <0.01]). We found some support of an interaction between elevation and average daily temperature (OR = 0.99, 95% CI [<−0.01, <0.01]). We did not analyze relationships of DSR with daily temperature range because daily temperature range was correlated with elevation.

**FIGURE 3:**
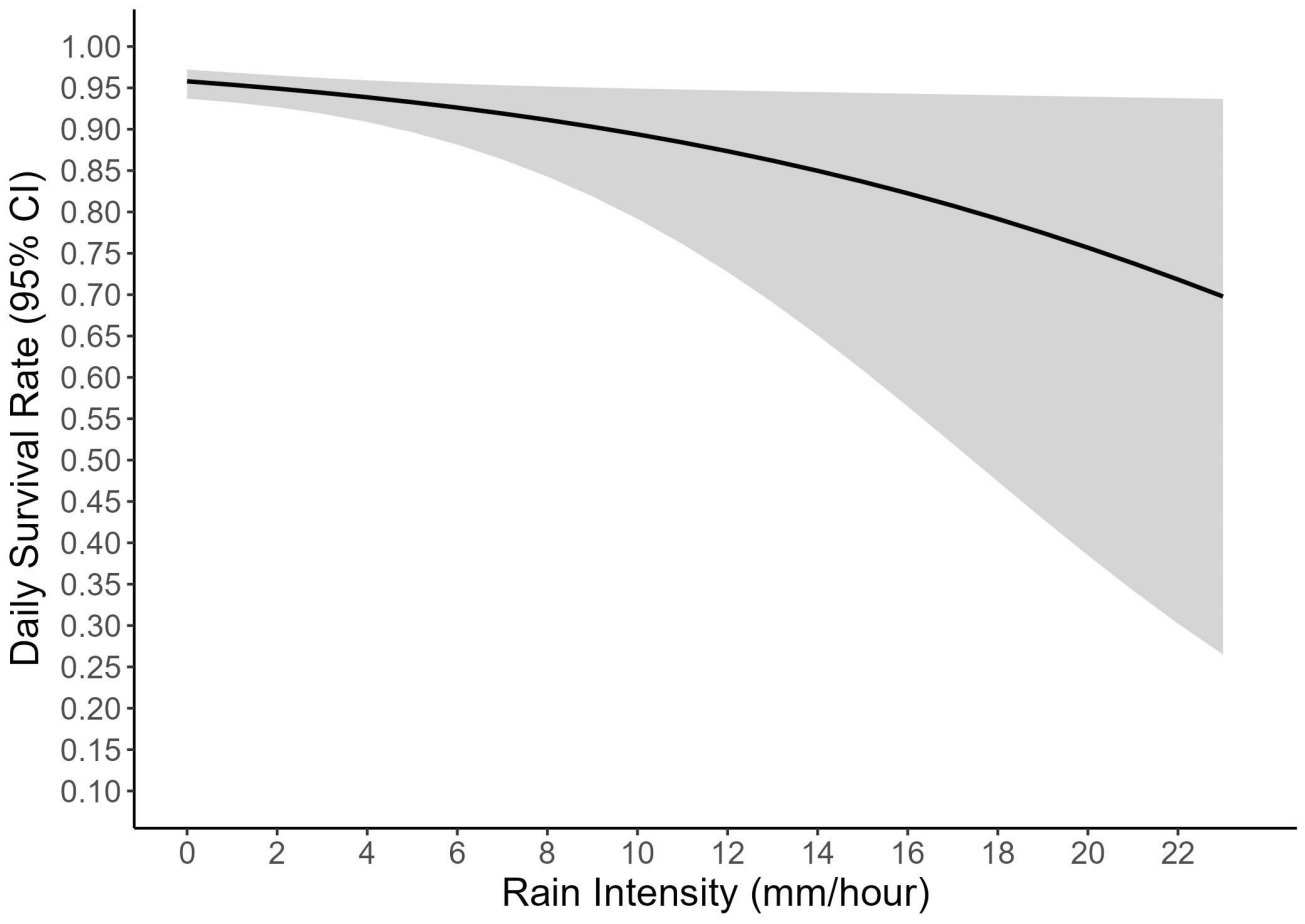
Estimated daily survival rate of Swainson's Thrush nests in the White Mountains, NH across rain intensity (mm/h) with 95% confidence intervals (shaded region). The model suggests that with every increase of 1 millimeter of rain, there is a 9% decrease in the odds of survival.

Rain intensity negatively affected DSR (OR = 0.91, 95% CI [−0.05, 0.13]; Figure 3), with each millimeter of rain/hour decreasing DSR by 9%. We did not find an effect of cumulative daily precipitation on DSR (OR = 1.01, 95% CI [−0.05, 0.12]), or interactions between rain intensity and cumulative daily precipitation on DSR (OR = 1.00, 95% CI [−0.02, 0.05]), rain intensity and nest age on DSR (OR = 1.00, 95% CI [−0.01, 0.02]), or between elevation and rain intensity on DSR (OR = 0.99, 95% CI [<−0.01, <0.01]).

The final stage of analysis using AICc model comparison revealed the top model included an interaction term between elevation and minimum daily temperature (Table 3). Two subsequent univariate models were within 4 ΔAICc values of the top model: linear time, followed by rain intensity (Table 3). Thus, we considered these models to reasonably fit the data similar to the top model. For brevity's sake, we included only the first 13 models in Table 3 from our complete model list (Table 1).

**TABLE 3:** A summary of nest survival model selection results using AICc (Akaike's Information Criterion) model comparison for Swainson's Thrush in the White Mountains, NH separated between all years (*n* = 58) and just 2019 and 2021 (*n =* 44).

Model (DSR~)	Parameters	AICc	ΔAICc	*w*i	Deviance
All years (2016, 2018, 2019, 2021)					
*TimeTrend*	2	189.34	0.00	0.34	185.32
TT	3	190.10	0.76	0.23	186.08
DSR (intercept only)	1	190.81	1.46	0.16	188.80
Year	2	191.48	2.41	0.11	187.46
Elev	2	192.18	2.84	0.08	188.16
NestAge	2	192.77	3.43	0.06	188.76
2019 and 2021 only					
**ElevMin**	4	151.49	0.00	0.45	143.42
*TimeTrend*	2	154.31	2.82	0.10	150.29
**Intensity**	2	154.98	3.48	0.07	150.96
TT	3	155.07	3.58	0.07	151.05
DSR (intercept only)	1	155.44	3.94	0.06	153.43
TempRange	2	155.93	4.43	0.04	151.91
Elev*MeanTemp	4	156.96	5.46	0.02	148.89
Elev	2	157.18	5.69	0.02	153.16
MinTemp	2	157.29	5.79	0.02	153.27
Init	2	157.33	5.86	0.02	153.31
MeanTemp	2	157.36	5.86	0.02	153.34
Precip	2	157.41	5.92	0.02	153.39
NestAge	2	157.43	5.93	0.02	153.41

*Note*: 95% CI significance is denoted when the model's name is in bold, 90% denoted in italics. Clarification of model names and effects can be referred to in Table 1.

**FIGURE 4:**
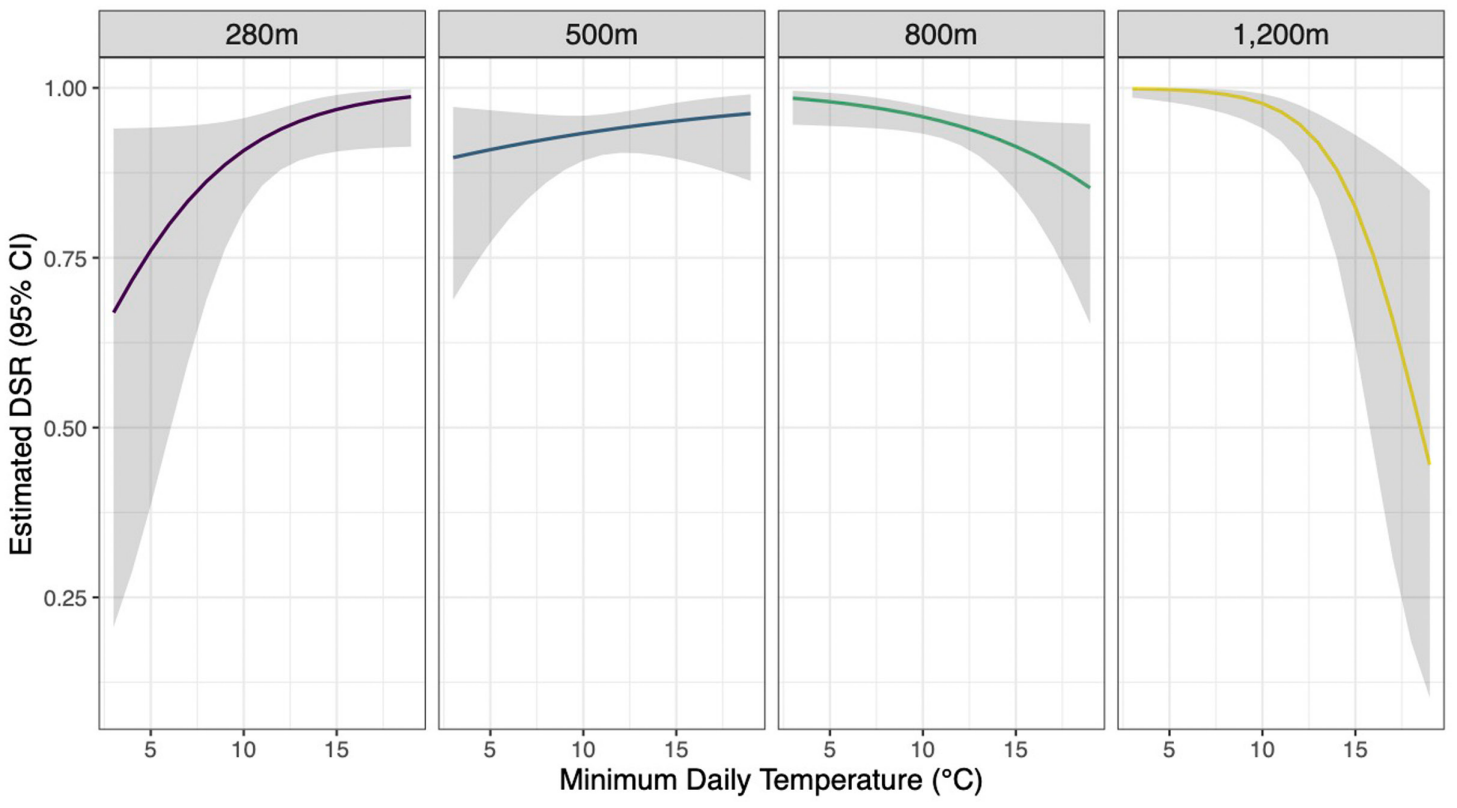
Estimated daily survival rate (DSR) of Swainson's Thrush nests in the White Mountains, NH across minimum daily temperature (°C) when elevation (m) is held constant, with 95% confidence intervals (shaded region). We obtained the probability of the beta estimate of the model and multiplied this value at 4 different elevations (280, 500, 800, and 1200 m) to find the slope. This figure illustrates that at low elevations (280 m), Swainson's Thrush nests have a lower DSR when minimum daily temperatures are low, and high‐elevation nests (1200 m) have a lower DSR when minimum daily temperatures are high.

To better understand the underlying interaction effect on DSR between covariates in our top model, we examined DSR across the range of minimum daily temperatures while holding elevation constant at four different levels (Figure 4). Swainson's Thrush nest DSR at low elevations (281 m) increased with increasing minimum daily temperatures. DSR at middle elevations (500 m and 800 m) remained relatively constant with increasing minimum daily temperatures and DSR at high elevations (1200 m) decreased with increasing minimum daily temperatures (Figure 4).

## 4 DISCUSSION

Our results demonstrate that within northeastern forests, nest survival of passerine birds may be negatively influenced by abiotic factors. Specifically, we found a negative interaction effect of minimum daily temperature and elevation on DSR (Figure 4). Similarly, we found that nest survival decreased during intense rain bouts, and with linear time, where nests had a lower probability of surviving later in the breeding season. These findings have implications for montane breeding birds as warmer temperatures and more frequent precipitation events are likely to occur due to climate change, both factors that have been documented to affect species' ranges in this region (Duclos et al., 2019; Murray et al., 2021; Westra et al., 2014).

Montane birds face several tradeoffs when breeding along an elevation gradient. For example, DeLuca (2013) documented blackpoll warblers had higher nesting success and overall higher annual fecundity at high‐elevation breeding sites. However, high‐elevation breeding pairs of dark‐eyed juncos (*Junco hyemalis*) produced offspring in better condition than their low‐elevation population, potentially because of higher parental investment in these conditions (Badyaev & Ghalambor, 2001; Bears et al., 2009). We predicted Swainson's Thrush nest survival would be positively related to elevation due to the lower predator numbers at high elevations and higher success from other documented literature (Boyle, 2008; Camfield et al., 2010; DeLuca, 2013). However, our model did not support this prediction. Another purpose for examining this effect was because nest depredation within the White Mountains has historically been affected by red squirrel (*Tamiasciurus hudsonicus*) densities on biennial cone cycles, creating challenges for nesting bird species when these fir trees produce mass crops (Hill, 2022; Townsend et al., 2015). Specifically, red squirrel densities negatively affected nest survival of American redstarts at a site approximately 64 km west of our study site, raising the possibility that high nest predation is likely during these fluctuating seed‐bearing tree years (Hubbard Brook Experimental Forest) (Sherry et al., 2015). Furthermore, predation from birds may be more active in warmer temperatures, potentially resulting in unusually higher predation events in warmer, mass crop years (Cox et al., 2013). If montane species' ranges continue to shift upslope, they may avoid the increased predation effects at lower elevations but consequently, will be at risk of high‐elevation abiotic influences (DeLuca & King, 2017; Van Tatenhove et al., 2019). Though we did not monitor predator density due to equipment and time constraints, further investigation of predator abundances and mass crops of fir trees along the elevation gradient would help inform how influential predation could be on nest survival of passerine species.

Extreme precipitation events have been shown to negatively influence nest survival of several species, including in great tits (*Parus major*), northern wheatears (*Oenanthe oenanthe*), and in mountain plovers (*Charadrius montanus*) (Bordjan & Tome, 2014; Dinsmore et al., 2002; Dreitz et al., 2012; Öberg et al., 2015; Schöll & Hille, 2020). Additionally, due to the orographic effect (i.e., colder high‐elevation air forcing clouds to release water), higher montane elevations are likely to receive more rain. Consistent with these prior studies, we found rain intensity negatively affected DSR (Table 2, Figure 3), suggesting that heavy rain may have negative consequences on nesting of Swainson's Thrushes. This is further supported by our observations during extreme precipitation events between 30 June–4 July 2021 (Figure 2b) that coincided with a noticeable pulse in nest failures (4 depredated, 4 abandoned). We examined interaction effects to evaluate if there were more nest failures after this rain event, and if nest age was affected by heavy rain events, but we did not find support for these relationships. Heavy rain may increase begging behavior of nestlings, and therefore, attract predators to the nest (Haff & Magrath, 2011; Leech & Leonard, 1997). Nests that failed between 30 June–4 July 2021 had nestlings ~5+ days old, and perhaps in addition to increased begging of hungry young, parents were unable to maintain their own energetic demands in addition to caring for their nestlings (Bordjan & Tome, 2014; Martin, 1987, 1995). Adults may have chosen to renest after the inclement weather passed, though this is risky in that fledglings born later in the season are less likely to survive (Shutler et al., 2006). Heavy precipitation events will become more frequent as the climate continues to warm, putting these montane breeding birds at risk of these effects (Trenberth, 2011; Westra et al., 2014).

Colder temperatures have been shown to negatively influence nest survival of high‐elevation breeding birds (Pierce et al., 2019). This can influence food availability by delaying spring insect emergence, especially at higher elevation sites where there may be persisting spring snow (Bears et al., 2009; Forrest & Thomson, 2011; Hahn et al., 2004). Conversely, warm temperatures enhance arthropod abundance and promote higher hatching and fledgling success in some breeding bird species (Martin, 1987; Reid et al., 2000; Reneerkens et al., 2011; Tulp & Schekkerman, 2008). In our system, the top model revealed that nests at low elevations (281 m) experienced lower DSR when minimum daily temperatures were low, but birds at high elevations showed the opposite pattern, experiencing lower DSR when minimum daily temperatures were high. Complementing our results, Dreitz et al. (2012) reported higher nest success in mountain plovers at high elevations when temperatures were cooler. Perhaps Swainson's Thrushes are not accustomed to the increased temperatures at high elevations and thus, nests were more likely to fail on hotter days. Exposure to increasingly warm temperatures may also lead to increased risk of nestling mortality rates due to the inability to regulate egg temperature and energy demands, prompting heat stress (Bourne et al., 2020; Carroll et al., 2018; Oswald et al., 2021). Individuals may choose nest sites in cooler areas at high elevations to prevent their nest from exposure to high temperatures, a behavior seen in other breeding birds (Northern bobwhite (*Colinus virginianus*) (Carroll et al., 2018); American robin (*Turdus migratorius*) (Ospina et al., 2018)). Other literature has reported warmer temperatures overall contribute positively to nest success and promote larger egg size, increased clutch size, and improved hatching and fledgling success (Hargrove & Rotenberry, 2011; Johnson, 2000; Lessells et al., 2002; Martin, 1987; Reid et al., 2000; Rotenberry & Wiens, 1991). However, warmer temperatures may prompt predators to be more active and thus, more frequent predation events could occur (Cox et al., 2013). Further investigation on direct effects of climate on breeding montane birds should be evaluated to better understand the mechanistic drivers of these climatic influences on nest survival.

Warming temperatures and inclement weather events reflect the changing climate within the White Mountains (Hamburg et al., 2013; IPCC, 2014; Seidel et al., 2009). These climatic effects are resulting in negative direct and indirect effects on species that breed in this region, causing elevational shifts in montane bird communities (DeLuca & King, 2017; Duclos et al., 2019; Van Tatenhove et al., 2019). Although previous literature has documented positive relationships with nest survival and warmer temperatures, these climatic effects can initiate major phenological changes and mismatches within bird populations (Hargrove & Rotenberry, 2011; Leech & Crick, 2007; Martin, 1987; Reid et al., 2000). This could be a disadvantage for single brood species, such as the Swainson's Thrush, who may rely on certain timing of events (i.e., insect emergence, habitat availability, food abundance) during the breeding season (Mack & Yong, 2020). It should be noted that a mismatch between nesting phenology and insect emergence was not documented at a nearby experimental forest within the White Mountains (black‐throated blue warblers, *Setophaga caerulescens* in Hubbard Brook Experimental Forest) (Lany et al., 2016). However, Swainson's Thrush breed at higher elevations and occupy sensitive habitat, perhaps putting them (and other high‐elevation breeding birds) at greater risk of climatic and phenological influences as a result of the warming temperatures.

Long‐term monitoring with the White Mountains has shown an increase in the frequency of precipitation events, which are likely to become more pronounced in the future (IPCC, 2014; Murray et al., 2021; Wason et al., 2017). Within this region, Duclos et al. (2019) reported that abundance of Swainson's Thrushes in this same study region was positively associated with precipitation across an elevational range of 319–1412 m. Invertebrate prey might be more accessible during precipitation events, and thus the effects of precipitation on Swanson's Thrushes may represent a tradeoff between DSR and food availability (Molyneux et al., 2018; Sullivan et al., 2016). In contrast, our results provide evidence that heavy rain and warmer minimum daily temperature at high elevations negatively influenced nest survival of Swainson's Thrush, which could have long‐term negative consequences to population trends (Hoekman et al., 2002; Wisdom & Mills, 1997).

This study documents evidence that Swainson's Thrush daily nest survival may be negatively affected by precipitation (rain intensity), as well as minimum daily temperature at high elevations, though a better mechanistic understanding of how climate directly affects nesting survival is needed. Several factors that were not measured in this study could contribute to nest survival, including habitat structure, predator abundance, and food availability. It is worth noting that other studies have assessed the effects of temporal and abiotic variables on nest survival and had considerably larger sample sizes than us and thus, could be one of the limitations this study faces (Dinsmore et al., 2002; Dreitz et al., 2012; Johnson et al., 2018; Pierce et al., 2019). Small effect sizes suggest weak support for these results, though our research is supported by other literature that documents these effects on nest survival. The warming climate will prompt more frequent rain bouts and shifts in plant and animal communities, exposing species to factors they are not accustomed to (Dunn & Winkler, 2010; Parmesan, 2007). Breeding birds will be at risk of these shifts, especially for species that occupy such restricted niches, like montane birds. Specifically, one species of concern in northeastern forests is the Bicknell's Thrush (*Catharus bicknelli*), a rare and vulnerable species that is facing population declines and has already been negatively impacted due to climatic effects (Hill et al., 2019; Lambert et al., 2008; Rodenhouse et al., 2008). Aggressive behavioral responses from Swainson's Thrush towards Bicknell's Thrush playback suggests that Swainson's Thrush may follow the “push” hypothesis and force Bicknell's Thrush out of their climatic niche, a likely occurrence due to species range shifts (DeLuca & King, 2017; Freeman & Montgomery, 2015). Our inspiration for this study was considered due to the vulnerability the Bicknell's Thrush faces, as well as the finding that Duclos et al. (2019) reported. As we continue to better understand the drivers that influence these sensitive montane species, this baseline knowledge will help inform land managers about how passerine birds and their population sizes will cope with the effects of climate change.

## AUTHOR CONTRIBUTIONS

**Sarah C. Deckel:** Conceptualization (equal); data curation (lead); formal analysis (lead); investigation (equal); methodology (equal); project administration (lead); software (equal); supervision (equal); validation (equal); visualization (equal); writing – original draft (lead). **William V. DeLuca:** Conceptualization (equal); formal analysis (supporting); investigation (equal); methodology (equal); supervision (equal); visualization (lead); writing – review and editing (equal). **Alexander R. Gerson:** Conceptualization (equal); formal analysis (supporting); funding acquisition (supporting); investigation (equal); methodology (equal); supervision (equal); validation (equal); visualization (equal); writing – review and editing (equal). **David I. King:** Conceptualization (lead); funding acquisition (lead); investigation (equal); methodology (equal); project administration (equal); resources (equal); supervision (equal); visualization (equal); writing – review and editing (equal).

## CONFLICT OF INTEREST STATEMENT

None declared.

## Supporting information


Appendix S1

## Data Availability

Raw data, supplementary materials, and R scripts can be found on Dryad (https://doi.org/10.5061/dryad.d51c5b073). Data includes nest survival and daily weather data, supplementary figures and tables, and an rMarkdown of script used to analyze DSR (Appendix S1).
