# Supplementary material for: Factors affecting the nesting success of Swainson's Thrush (Catharus ustulatus) along an elevational gradient
Source: Ecol Evol. 2024 Jan 17;14(1):e10738. doi: 10.1002/ece3.10738 (PMC10792399; doi:10.1002/ece3.10738)
Supplement: Supplementary file 1 — Appendix S1 [file ECE3-14-e10738-s001.zip › Supplementary_Material_SWTHmanuscript(1).docx]

**Supplementary Material**

**Methodology**

-Random Forest (RF) Algorithm to estimate missing values for 34 days

-Dummy variable (0 or 1) randomly assigned to 34 days

*Data continues to 8/3/21

**Data**

Missing values from 1 rain gauge (“low Jeff” = 500m)

*1 July – 3 August 2021*

(34 days total)

**Results**

-Impute values from RF on days where dummy variable = 1

-Confirm fit using performance (HK value) and error metrics (NRMSE)


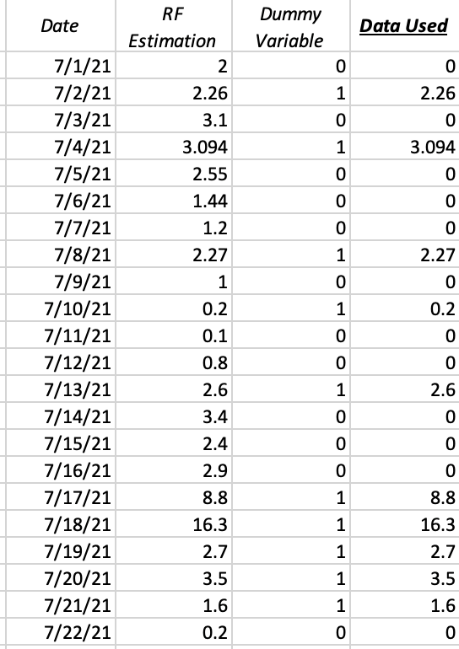


HK = 0.39

NRMSE = 0.078


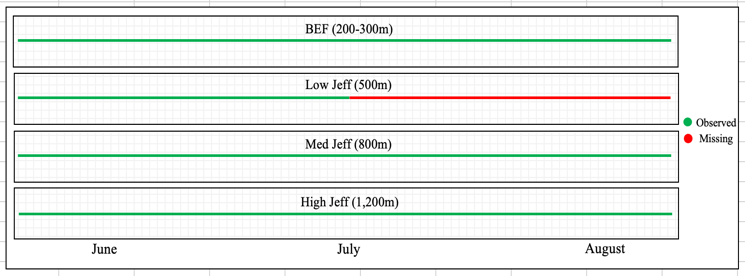


*Supplementary Figure 1.* A guide to the methodology used to estimate missing precipitation data. Once we identified how much data we needed to estimate, we used the Random Forest (RF) algorithm to predict/estimate precipitation data in the imputation. A dummy variable was randomly assigned to the day to determine if we did (1) or didn’t (0) include the estimated imputations from the RF. Performance and error metrics were included: (HK = Hanssen and Kuipers; NRMSE = normalized root error mean squared error). This figure is a modification derived from Aguilla, Guardiola-Albert and Serrano-Hidalgo (2020), where we also obtained the recommendation for the methodology used.

Supplementary Figure 2. Rain intensity (millimeters per hour) across the season (ordinal date) in 2019 (A) and 2021 (B) within the White Mountains, NH. Data is faceted by elevation bin, and represents “BEF” (200m), “Low Jeff” (500m), “Med Jeff” (800m) and “High Jeff” (1,200m).

**A**

*
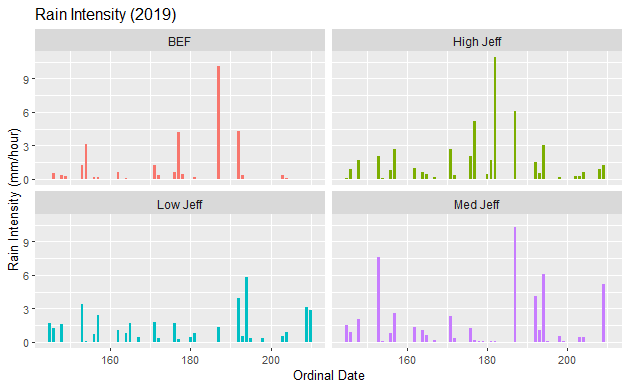
*

*
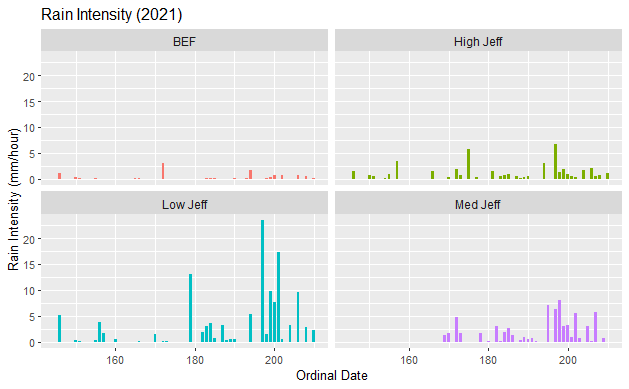
*

**B**

*Supplementary Table 1.* A list of all multivariate models that were analyzed. The global model is the last listed model (# 36). See Table 1 (in manuscript) for interpretation of variable abbreviation. Variables that exhibited correlation were not included in the same model (elevation with daily temperature range, and elevation with minimum daily temperature).

| Candidate model (DSR~) |  |
| --- | --- |
| 1. Elev + TimeTrend + Precip 2. Elev + TimeTrend + Init 3. Elev + TimeTrend + NestAge 4. Elev + TimeTrend + MeanTemp | |
| 1. Elev + TimeTrend + Intensity 2. Elev * TimeTrend + Precip 3. Elev * TimeTrend + Init 4. Elev * TimeTrend + NestAge 5. Elev * TimeTrend + MeanTemp 6. Elev * TimeTrend + Intensity 7. Elev * Precip + Intensity 8. Elev * NestAge + Intensity 9. Elev * Initiation + Intensity 10. MeanTemp * Intensity 11. MeanTemp + Intensity 12. MeanTemp * Intensity + NestAge 13. MeanTemp * Intensity + TimeTrend 14. MeanTemp * Intensity + Init 15. MeanTemp * Precip + NestAge 16. MeanTemp * Precip + TimeTrend 17. MinTemp * Intensity + NestAge 18. MinTemp * Intensity + TimeTrend 19. MinTemp * Intensity + Init 20. MinTemp * Precip + TimeTrend 21. MinTemp * Precip + Nestage 22. MinTemp * Precip + Init 23. MinTemp + Intensity 24. MinTemp * Intensity 25. TempRange * Intensity + Init 26. TempRange * Intensity + TimeTrend 27. TempRange * TimeTrend + Intensity 28. TempRange * Init + Intensity 29. TempRange * Precip + Init 30. TempRange * Precip + TimeTrend 31. TempRange * Precip + NestAge 32. Elev * Intensity + NestAge + Precip + Init + TimeTrend + MeanTemp | |
